# *De novo* assembly of the complete mitochondrial genome of pepino (*Solanum muricatum*) using PacBio HiFi sequencing: insights into structure, phylogenetic implications, and RNA editing

**DOI:** 10.1186/s12870-024-04978-w

**Published:** 2024-05-04

**Authors:** Ziwei Li, Jiaxun Liu, Mingtai Liang, Yanbing Guo, Xia Chen, Hongzhi Wu, Shoulin Jin

**Affiliations:** 1 Yunnan Agricultural University, Kunming, Yunnan 650201 China; 2 Horticultural Research Institute Yunnan Academy of Agricultural Sciences, Kunming, Yunnan 650205 China

**Keywords:** *Solanum muricatum*, HiFi sequencing, Mitochondrial genome, Phylogenetic relationship, RNA editing

## Abstract

**Background:**

*Solanum muricatum* is an emerging horticultural fruit crop with rich nutritional and antioxidant properties. Although the chromosome-scale genome of this species has been sequenced, its mitochondrial genome sequence has not been reported to date.

**Results:**

PacBio HiFi sequencing was used to assemble the circular mitogenome of *S. muricatum*, which was 433,466 bp in length. In total, 38 protein-coding, 19 tRNA, and 3 rRNA genes were annotated. The reticulate mitochondrial conformations with multiple junctions were verified by polymerase chain reaction, and codon usage, sequence repeats, and gene migration from chloroplast to mitochondrial genome were determined. A collinearity analysis of eight *Solanum* mitogenomes revealed high structural variability. Overall, 585 RNA editing sites in protein coding genes were identified based on RNA-seq data. Among them, *mttB* was the most frequently edited (52 times), followed by *ccmB* (46 times). A phylogenetic analysis based on the *S. muricatum* mitogenome and those of 39 other taxa (including 25 *Solanaceae* species) revealed the evolutionary and taxonomic status of *S. muricatum*.

**Conclusions:**

We provide the first report of the assembled and annotated *S. muricatum* mitogenome. This information will help to lay the groundwork for future research on the evolutionary biology of *Solanaceae* species. Furthermore, the results will assist the development of molecular breeding strategies for *S. muricatum* based on the most beneficial agronomic traits of this species.

**Supplementary Information:**

The online version contains supplementary material available at 10.1186/s12870-024-04978-w.

## Introduction

Mitochondria are semi-autonomous organelles found in almost all eukaryotic cells. According to the theory of endosymbiosis, mitochondria are derived from endosymbiotic α-proteobacteria [1]. The plant mitochondrial (mt) genome has become an important tool in the study of species origins, genetic diversity, and evolution [2–5]. In plant cells, mitochondria synthesize ATP through the tricarboxylic acid cycle and oxidative phosphorylation, which in turn provides energy for plant growth, development, and reproduction [6, 7]. Moreover, recent studies have documented that plant mitochondria are closely related to cytoplasmic male sterility, disease resistance, and plant growth vigor [8–10]. In contrast to the stable size and gene content of chloroplast (cp.) genomes, plant mt genomes vary widely in complexity [11–13]. For example, the smallest mt genome reported to date contains 66 kb (*Viscum scurruloideum*) [14], while the largest reaches 11.7 Mb in size (*Larix sibirica*) [15]. Furthermore, apart from the common monocyclic structures, plant mt genome structures can be linear, multibranched, and polycyclic [12, 13], suggesting complexity in the assembly of the plant mt genome.

Pepino (*Solanum muricatum* Aiton), a member of the Solanaceae family, which has ca. 2300 species in 95 genera, is a perennial herbaceous domesticated crop originating from the Andes in South America [16, 17]. In recent years, pepino has gained recognition world-wide due to its aromatic, juicy, and nutritious fruits, which are rich in potassium, selenium, and vitamin C. Pepino is, therefore, emerging as a promising horticultural fruit crop with human health benefits [18]. The traits and color of the fruits vary according to the cultivar, but those most commonly grown produce fruits with golden-yellow skin marked with purple stripes at maturity and yellow flesh that is aromatic, slightly sweet, and juicy [19, 20]. According to the sweetness/acidity characteristics of the cultivar, pepino can be consumed in salads, as a fresh fruit eaten directly, or as an ingredient in desserts, juices, or purees [21, 22]. In addition, pepino plants have excellent antioxidant, antidiabetic, anti-inflammatory, and anti-tumor activities [23–29]. Intensive research has been carried out on pepino to provide information about its abiotic and biotic stresses [30–34], plant photomorphogenesis [35, 36], fruit aroma and flavor [37–40], genetic diversity [41, 42], transcriptome [43], metabolome [40, 44, 45], and genome [46]. However, the dissection of biological functions of key genes for mitochondria-related traits is extremely challenging due to the absent of pepino mt genome.

With the rapid development of sequencing technology, especially the emergence of PacBio HiFi technologies that consider both read length and accuracy, numerous plant mt genome sequences have been reported. According to the NCBI, as of April 2023, a total of 602 plant mt genomes, and 10,479 cp. genomes have been released (https://www.ncbi.nlm.nih.gov/genome/browse#!/organelles/), although no information about the mt genome of *S. muricatum* has been reported to date. Up to now, more than fifteen mt genomes of *Solanum* species available on NCBI, including *Solanum lycopersicum*, *Solanum tuberosum*, *Solanum pennellii*, *Solanum melongena*, *Solanum bukasovii*, *Solanum aethiopicum*, *Solanum okadae*, *Solanum phureja*, *Solanum chaucha*, *Solanum ahanhuiri*, *Solanum stenotomum*, *Solanum wrightii*, *Solanum sisymbriifolium*, *Solanum* x *juzepczukii*, and *Solanum* x *curtilobum*, which has greatly enriched the genetic resources of the genus *Solanum* and provided breeders with a wealth of genetic data. Nevertheless, the mt genomes of most *Solanum* species have not been systematically studied, which severely constrained our study of mt genome evolution in this species.

In this study, we assembled the complete mt genome of pepino for the first time using PacBio HiFi data and conducted a holistic analysis of the genome structure, gene content, codon preference, repetitive sequences, RNA editing, phylogenetic relationships, and mitogenomic synteny. We then resolved the reticular mitochondrial conformation with multiple junctions and further verified the junctions using PCR. Furthermore, the cp. genome was assembled using Illumina data and gene transfers between the cp. and mt genomes were investigated. RNA editing sites were detected based on RNA-seq data from the mt genome. These results provided a solid foundation for developing genetic engineering strategies and elucidating the functional molecular mechanisms of mt genes in this versatile horticultural fruit crop.

## Results

### General features of the pepino mitogenome

**Fig. 1:**
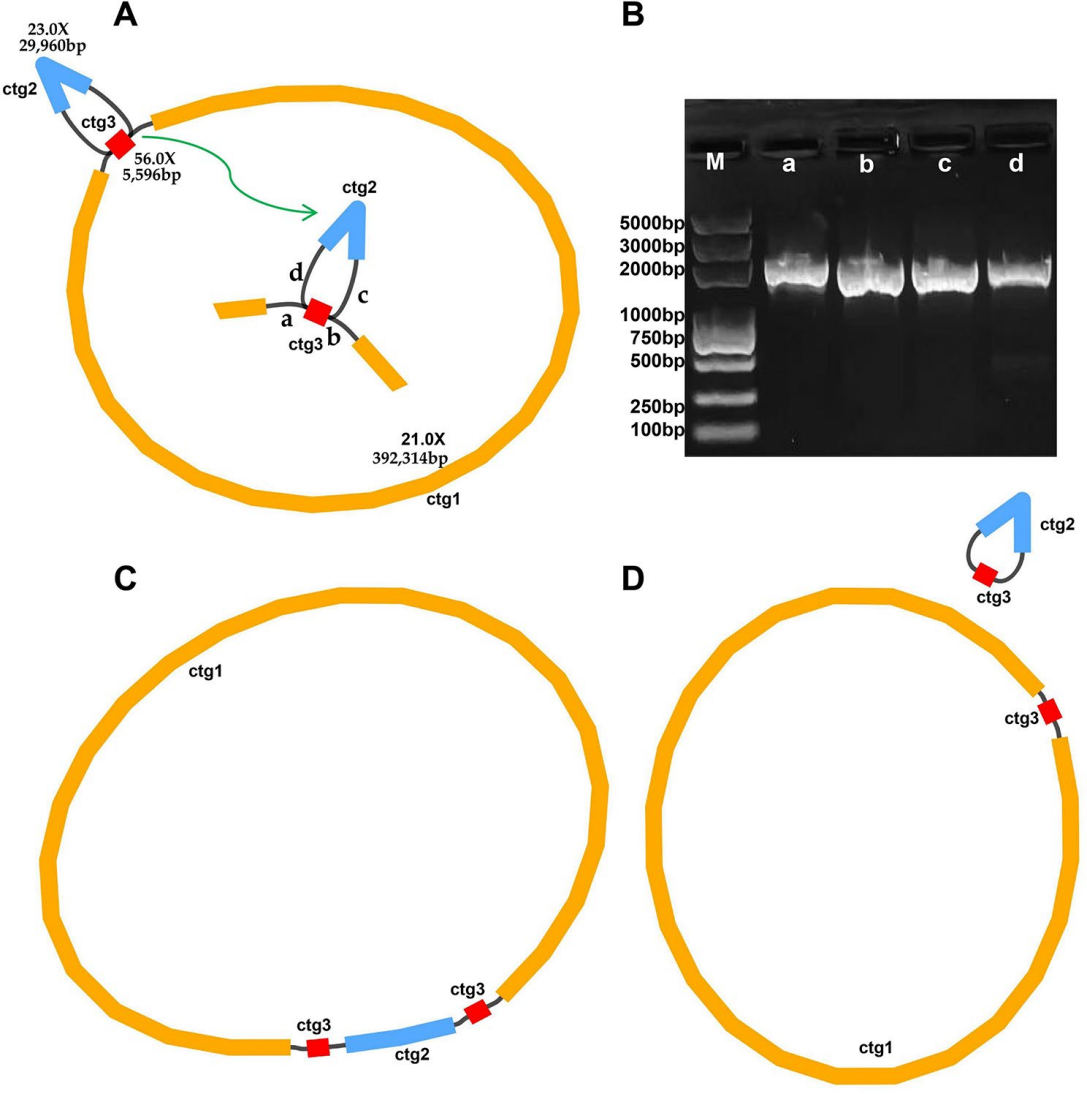
Mitogenome structure of pepino accession generated using Bandage software. (**A**) Draft of the mitochondrial (mt) genome assembly. At the center, a, b, c, and d represent the connections of ctg1-ctg3, ctg3-ctg1, ctg3-ctg2, and ctg2-ctg3, respectively. (**B**) PCR amplification to verify all four linkages in the *S. muricatum* mt genome conformation. The numbers above each lane of the gel refer to linkages spanned by the primers with respect to the contig. (**C**) Master circular structure of *S. muricatum* mt genome. (**D**) Recombinant structure of the mt genome of *S. muricatum*

The pepino mt genome sketch was assembled based on long-reads data and visualized using Bandage software (v0.8.1) (Fig. 1). We found that the unitig graph was mediated by one repeat sequence containing 5,596 bp, named ctg3 (Fig. 1A). To validate the key branch nodes, we exported the relevant sequences and mapped them to the long-reads data using BLASTn. The strategy was as follows: when two sequences connected along a black line appeared head-to-tail on the same long-read, the long-read was identified as supporting the interconnection of the two sequences; where there were multiple different connections on branch nodes, connections supported by more long-reads were preferred; and red nodes (Fig. 1A) represented potential repeat sequences that may appear multiple times in the genome. Using this strategy, we obtained a circular contig containing 433,466 bp, with a GC content of 44.79% (Fig. 1C; Table 1). The solution pathway can be seen in Table S1. The accuracy of the mitogenome assembly was confirmed by mapping the PacBio HiFi long reads (average 147-fold depth) onto the assembly (Fig. S1, Table S2). Notably, the repeat sequence (node ctg3) may mediate genomic recombination and form two small ring structures (Fig. 1D). Subsequently, four contig connections (a, b, c and d) were further verified by PCR to confirm that the expected length from the assembly matched the PCR product length (Fig. 1B, Fig. S2, and Table S3).

**Fig. 2:**
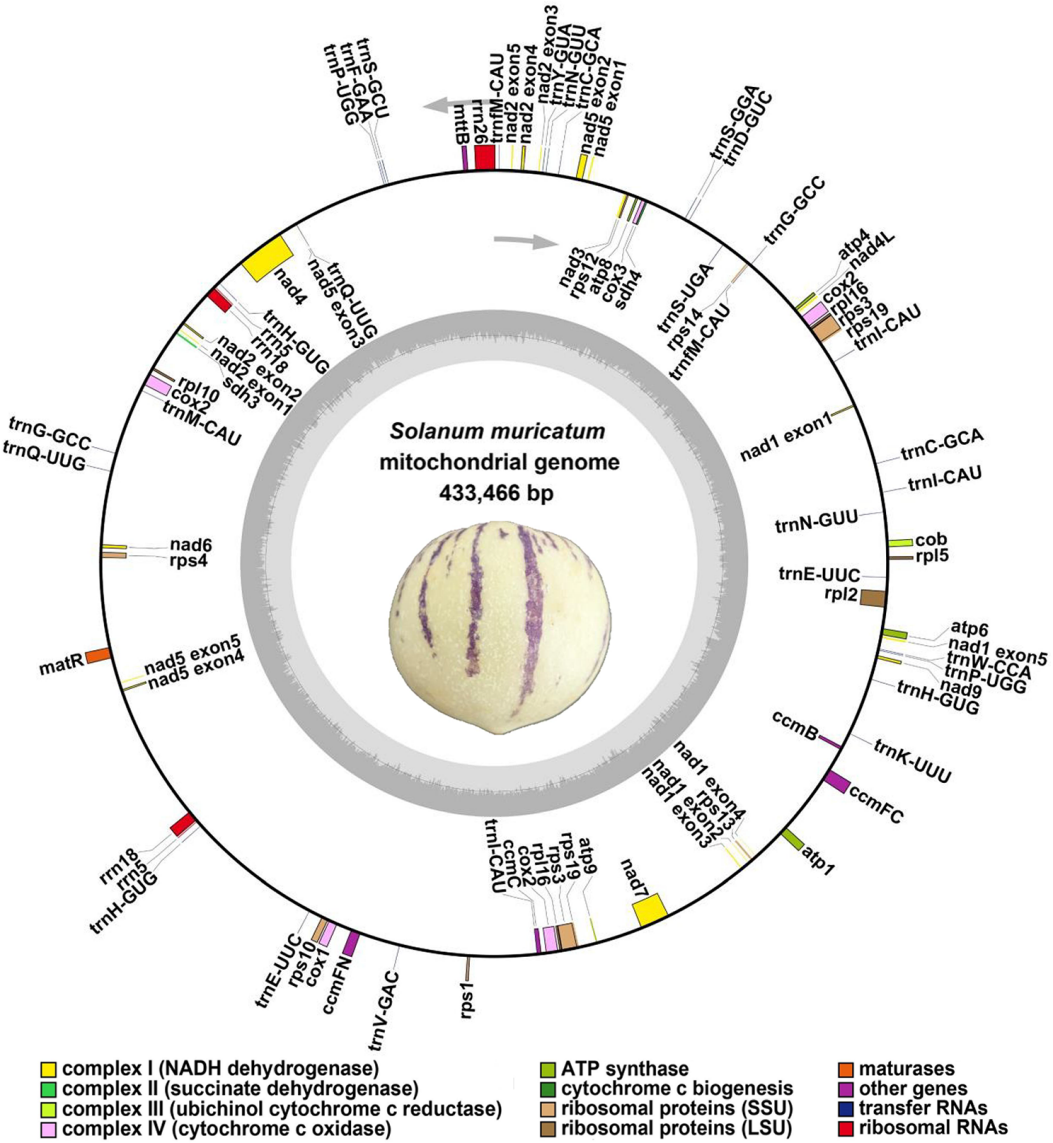
Map of the *S. muricatum* mitogenome. Clockwise- and counterclockwise-transcribed genomic features are drawn inside and outside the circles, respectively. Colors are used to distinguish genes of different functional groups

We annotated the mt genome of pepino, and the categorization of genes is shown in Fig. 2; Table 2. The pepino mt genome contained 60 annotated genes, namely 24 unique core genes, 14 variable genes, 19 tRNA and 3 rRNA genes. The core genes consisted of five ATP synthase genes (*atp1*, *atp4*, *atp6*, *atp8*, and *atp9*), nine NADH dehydrogenase genes (*nad1*, *nad2*, *nad3*, *nad4*, *nad4L*, *nad5*, *nad6*, *nad7*, and *nad9*), four cytochrome C biogenesis genes (*ccmB*, *ccmC*, *ccmFC*, and *ccmFn*), three cytochrome C oxidase genes (*cox1*, *cox2*, and *cox3*), a transport membrane protein (*mttB*), a maturase (*matR*), and ubiquinol cytochrome c reductase (*cob)*. The variable genes consisted of four large subunits of ribosome proteins (*rpl2*, *rpl5*, *rpl10*, and *rpl16*), eight small subunits of ribosome proteins (*rps1*, *rps3*, *rps4*, *rps10*, *rps12*, *rps13*, *rps14*, and *rps19*), and two respiratory genes (*sdh3*, and *sdh4*). Interestingly, three copies of *cox2* were found. Additionally, three variable genes, nine tRNA, and two rRNA genes located in repeat sequences were found to be present in two or three copies (*rpl16*, *rps3*, *rps19*, *trnC-GCA*, *trnE-UUC*, *trnfM-CAU*, *trnG-GCC*, *trnH-GUG*, *trnI-CAU*, *trnN-GUU*, *trnP-UGG*, *trnQ-UUG*, *rrn5*, and *rrn18*).

**Table 1:** Basic information about the *S. muricatum* mt genome

Type	mt genome
Structure	circular
Circular molecular number	1
Total length	433,466 bp
GC content	44.79%

**Table 2:** Gene composition of the pepino mitogenome

Group of genes	Name of genes
ATP synthase	*atp1*, *atp4*, *atp6*, *atp8*, *atp9*
NADH dehydrogenase	*nad1*, *nad2*, *nad3*, *nad4*, *nad4L*, *nad5*, *nad6*, *nad7*, *nad9*
Cytochrome c biogenesis	*cob*
Ubiquinol cytochrome c reductase	*ccmB*, *ccmC*, *ccmFC*, *ccmFN*
Cytochrome c oxidase	*cox1*, *cox2* (3), *cox3*
Maturases	*matR*
Transport membrane protein	*mttB*
Large subunit of ribosome	*rpl2*, *rpl5*, *rpl10*, *rpl16* (2)
Small subunit of ribosome	*rps1*, *rps3* (2), *rps4*, *rps10*, *rps12*, *rps13*, *rps14*, *rps19* (2)
Succinate dehydrogenase	*sdh3*, *sdh4*
Ribosome RNA	*rrn5* (2), *rrn18* (2), *rrn26*
Transfer RNA	*trnC-GCA* (2), *trnD-GUC*, *trnE-UUC*(2), *trnF-GAA*, *trnfM-CAU* (2), *trnG-GCC*(2), *trnH-GUG* (3), *trnI-CAU*(3), *trnK-UUU*, *trnM-CAU*, *trnN-GUU* (2), *trnP-UGG* (2), *trnQ-UUG* (2), *trnS-GCU*, *trnS-GGA*, *trnS-UGA*, *trnV-GAC*, *trnW-CCA*, *trnY-GUA*

*Note* The numbers in parentheses represent gene copy numbers

### Protein coding gene codon usage

**Fig. 3:**
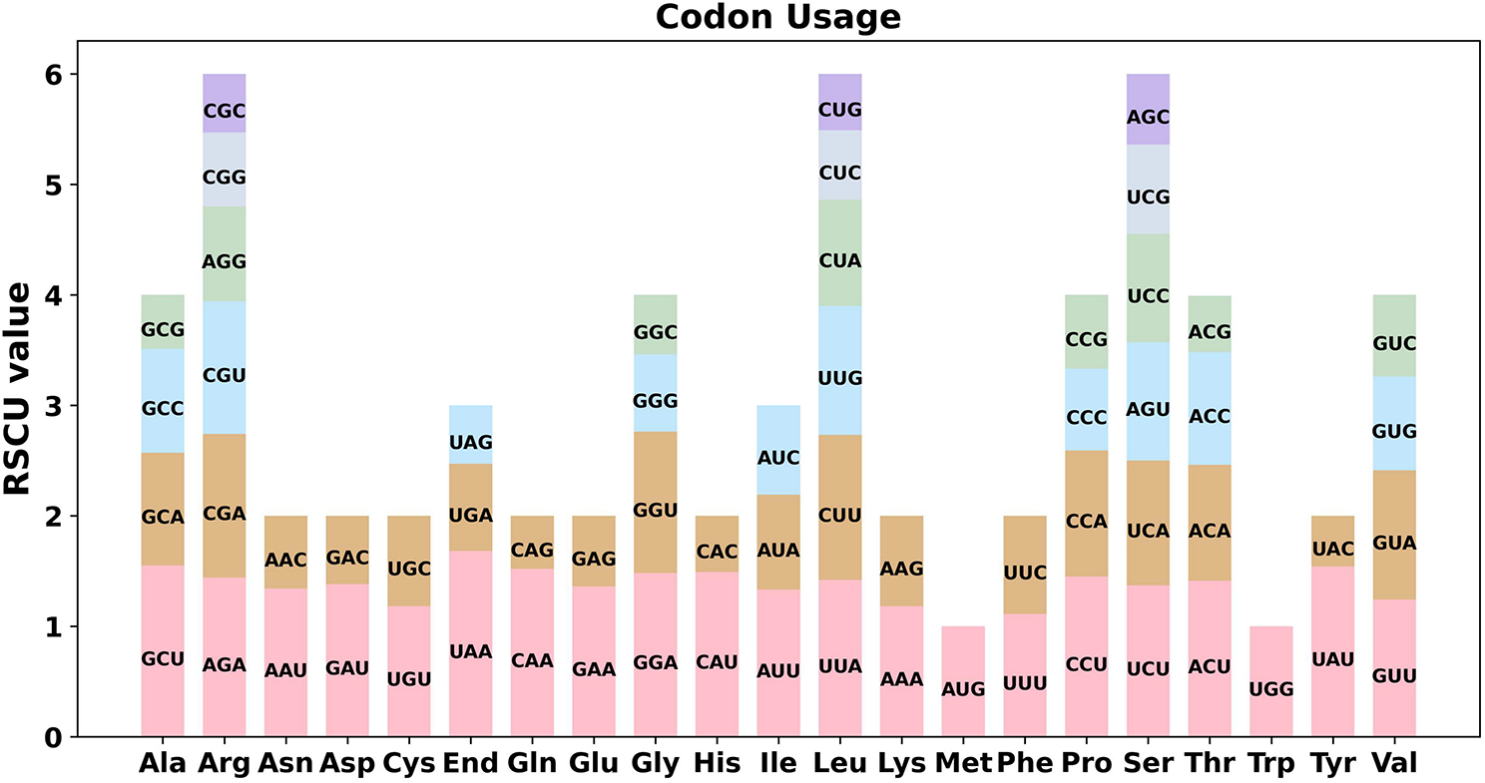
Pepino mitogenome codon preferences. RSCU = relative synonymous codon usage

The eukaryotic genome contained 64 codons that encode 20 amino acids and three stop codons. All amino acids except Met and Try were encoded by multiple codons. There were large differences in genome codon usage across species due to the degeneracy of codons. Herein, the 38 protein coding genes (PCGs) in the mt genome of *S. muricatum* were analyzed for codon preference, and the use of codons for individual amino acids is shown in Table S4. Codons with a relative synonymous codon usage (RSCU) value > 1 are considered to be used preferentially by amino acids. As shown in Fig. 3, except for the start codons AUG and UGG (Try), both of which had RSCU values of 1, there was also a general codon usage preference for mt PCGs. For example, the termination codon had a high preference for the use of UAA, which had the highest RSCU value among mt PCGs at 1.68. Next, ALA had a preference for GCU, with an RSCU value of 1.55. This preference may have resulted from the gradual development of a relative equilibrium in the pepino over a long period of evolutionary selection.

### *S. Muricatum* mitogenome repeats

**Fig. 4:**
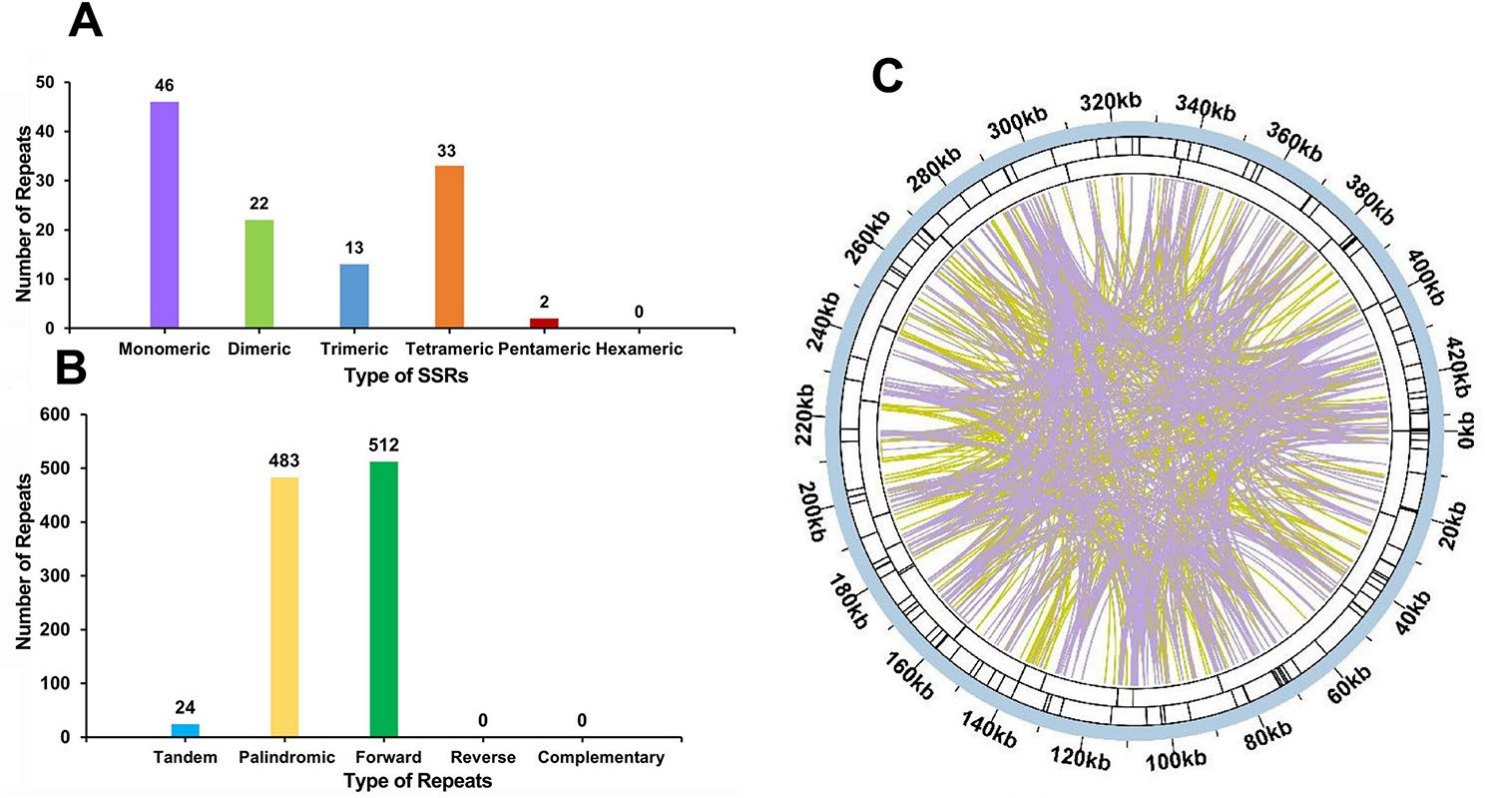
Repeat sequences in the pepino mt genome. (**A**) Type and number of simple sequence repeats (SSRs). The purple, green, blue, orange, and red colors indicate monomeric, dimeric, trimeric, tetrameric, and pentameric SSRs, respectively. (**B**) Type and number of repeats. The blue, yellow, and green colors indicate tandem, palindromic, and forward repeats, respectively. (**C**) The inner circle shows the dispersed repeats, with purple representing palindromic repeats and yellow representing forward repeats. The two outer circles show tandem repeats and SSRs as short bars, respectively

A total of 116 simple sequence repeats (SSRs) were detected in the pepino mt genome, among which monomeric and dimeric SSRs accounted for 58.62% of the total (Fig. 4A and C, Table S5). Thymine (T) monomeric repeats accounted for 54.35% (25) of the 46 monomeric SSRs. However, no hexameric SSR was detected in the mt genome of pepino.

Tandem repeats, also known as satellite DNA, are widespread in eukaryotic genomes and prokaryotes and form core repeating units of around 7 − 200 bases repeated multiple times in tandem. As shown in Table S6 and Figs. 4B and C and 24 tandem repeats in the mt genome with a match of more than 75% and 12–45 bp in length were detected.

The dispersed repeats in the mt genome of pepino were examined. A total of 995 pairs of repeats with lengths ≥ 30 bp were found, including 483 pairs of palindromic repeats and 512 pairs of forward repeats, but no reverse or complementary repeats were detected (Fig. 4B and C). The longest palindromic repeat was 8,353 bp, while the longest forward repeat was 5,637 bp, in length (Table S7).

### Plastid DNA insertion in mitogenome

**Fig. 5:**
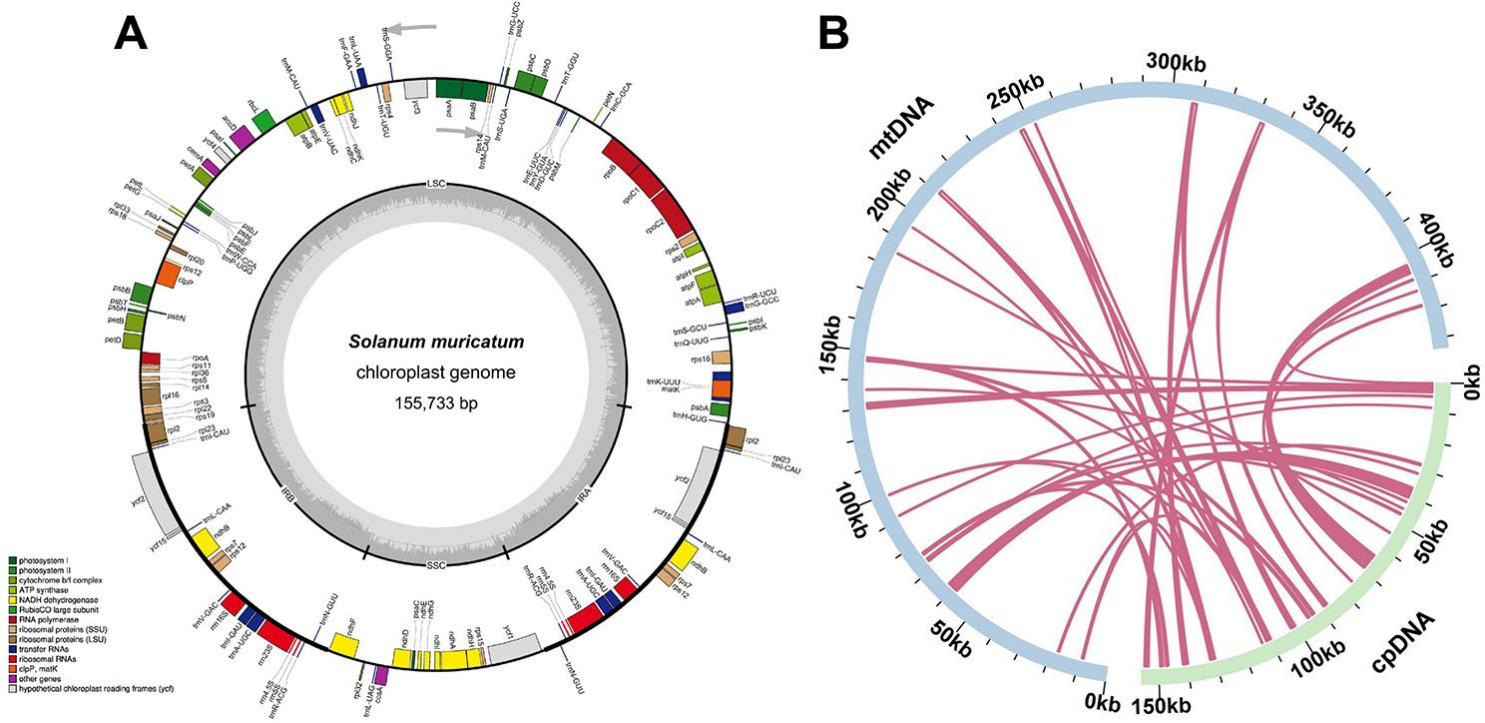
Genome map of the *S. muricatum* chloroplast and sequence migration. (**A**) *S. muricatum* chloroplast (cp.) genome map. Genes residing inside and outside of the outer circle are in the forward and reverse directions, respectively. The dark and light gray bars in the inner circle denote the G + C and A + T contents, respectively. **(B**) Schematic of 33 MTPTs of *S. muricatum.* The blue arc represents the mitochondrial (mt) genome, and the green arc represents the cp. genome. The pink lines between the arcs correspond to homologous genomic segments

Here, we assembled and annotated the pepino cp. genome, which contained 155,733 bp and 132 annotated genes, as shown in Fig. 5A. Subsequently, we conducted homologous fragment analysis between the pepino mt genome and cp. genome based on the BLASTn program. In total, 33 homologous DNA fragments (Mitochondrial plastid DNAs, MTPTs) were observed (Fig. 5B, Table S8), among which 12 were over 1,000 bp, and the longest was 3,651 bp, while the shortest fragment was only 29 bp in length. The total length of these 33 fragments was 20,759 bp and occupied 4.79% of the mitogenome. We annotated 33 homologous fragments and found 19 complete genes, including 8 PCGs (*petA*, *petG*, *petL*, *psbJ*, *psbL*, *psbZ*, *rpl23*, and *rps14*) and 11 tRNA genes (*trnC-GCA*, *trnD-GUC*, *trnG-UCC*, *trnH-GUG*, *trnI-CAU*, *trnM-CAU*, *trnN-GUU*, *trnP-UGG*, *trnS-GGA*, *trnV-GAC*, and *trnW-CCA*). Additionally, our results revealed that some PCGs, i.e., *ndhF*, *psaB*, *ycf2*, *ycf3*, *rpl2*, *psbA*, *psbB*, *psbF*, and *cemA*, migrated from the cp. genome to the mt genome in pepino (Table S8), and most of them lost their integrity during evolution.

### Phylogenetic evolution and sequence collinearity

**Fig. 6:**
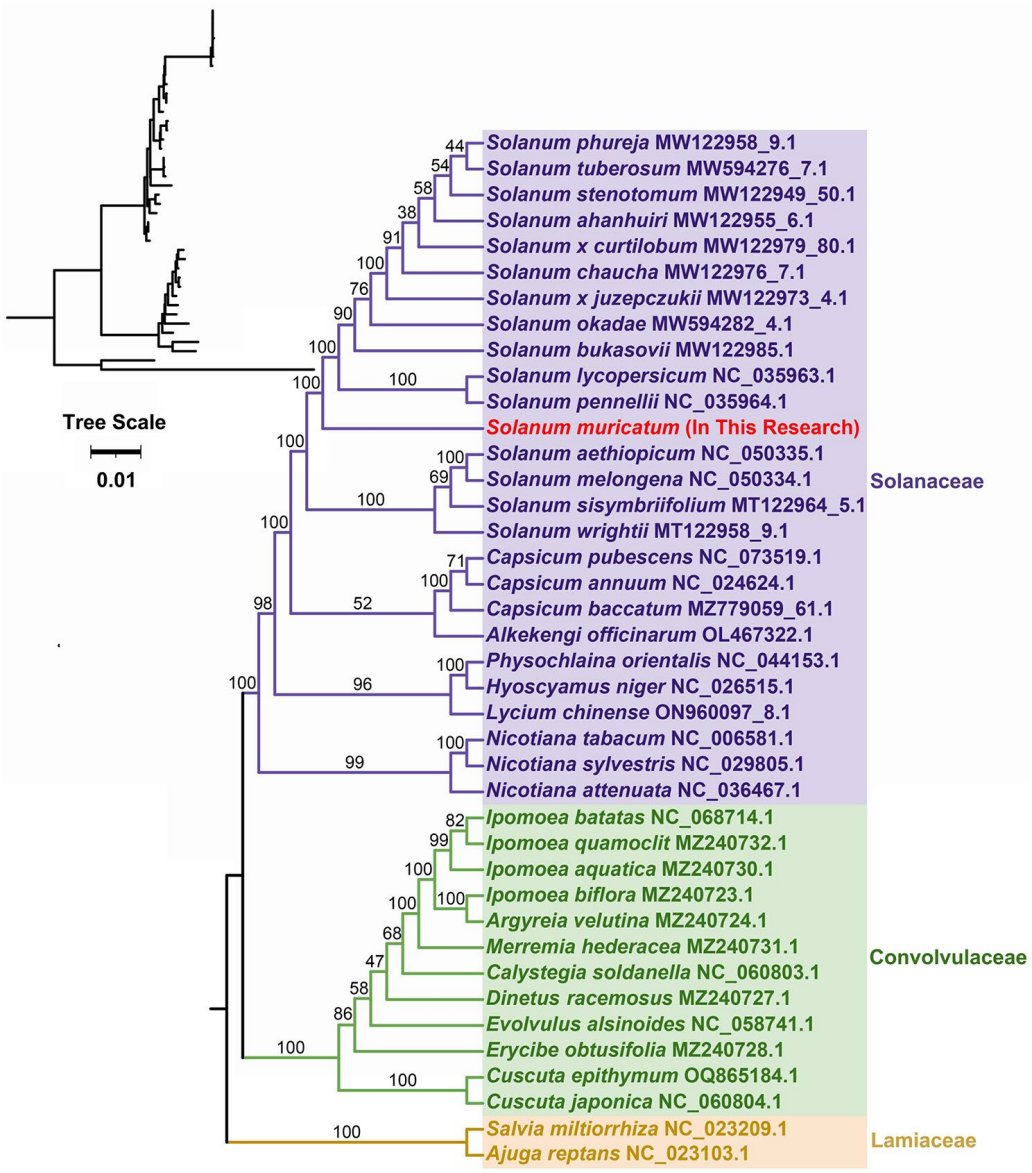
Phylogenetic analysis of the *S. muricatum* mitochondrial (mt) genome based on 15 conserved mt protein coding genes (PCGs). Numbers related to the branches are bootstrap-supported values

To determine the evolutionary status of *S. muricatum*, 49 mitogenome data sets from species in the Solanaceae (25), Convolvulaceae (12), and Lamiaceae (2) were obtained from the NCBI genome database (Table S9). Based on 15 single-copy homologous genes shared by 40 species (*atp6*, *atp8*, *atp9*, *ccmFC*, *cob*, *cox1*, *cox2*, *matR*, *nad2*, *nad3*, *nad4*, *nad5*, *nad6*, *rpl2*, and *rps12*), a maximum likelihood (ML) tree was constructed with *Salvia miltiorrhiza* (NC_023209.1) and *Ajuga reptans* (NC_023103.1) as outgroups. As shown in Fig. 6, the taxa from three families (Solanaceae, Convolvulaceae, and Lamiaceae) were well clustered, which is consistent with the results of the APG IV classification system. In the cluster of the Solanaceae family, species from the Solanum, Capsicum, and Nicotiana genera were well grouped. The results also reflected that *S. muricatum* was more closely related to tomato (*Solanum lycopersicum* and *Solanum pennellii*), while distantly related to potato (*Solanum tuberosum*).

**Fig. 7:**
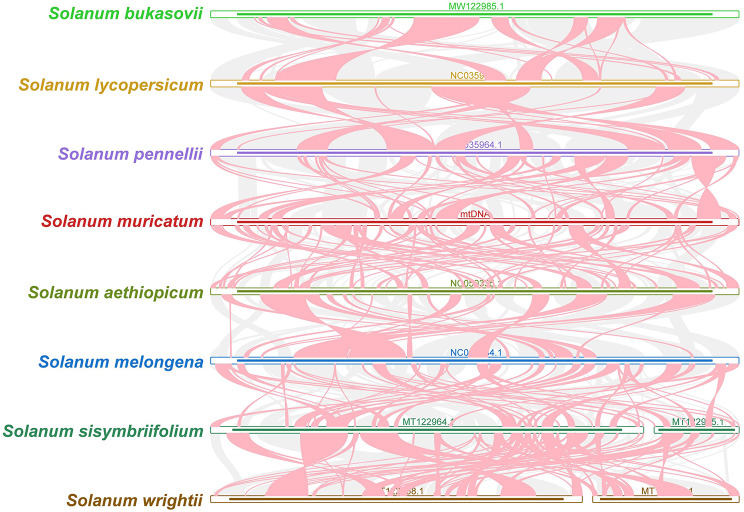
Mitogenome synteny. The red areas indicate inversion, grey areas indicate good homology

Genomic rearrangements due to repeat sequences is a primary cause of mt genome evolution in plants. Many homologous co-linear blocks were detected between *S. muricatum* and closely related species (Fig. 7, Table S10). The results indicated the presence of co-linear blocks with inconsistent orders of arrangement among individual mitogenomes; that is, the *S. muricatum* mitogenome has experienced massive genomic rearrangements with closely related species, and the structure of the mt genome is extremely unconserved.

### RNA editing sites in the pepino mitogenome

**Fig. 8:**
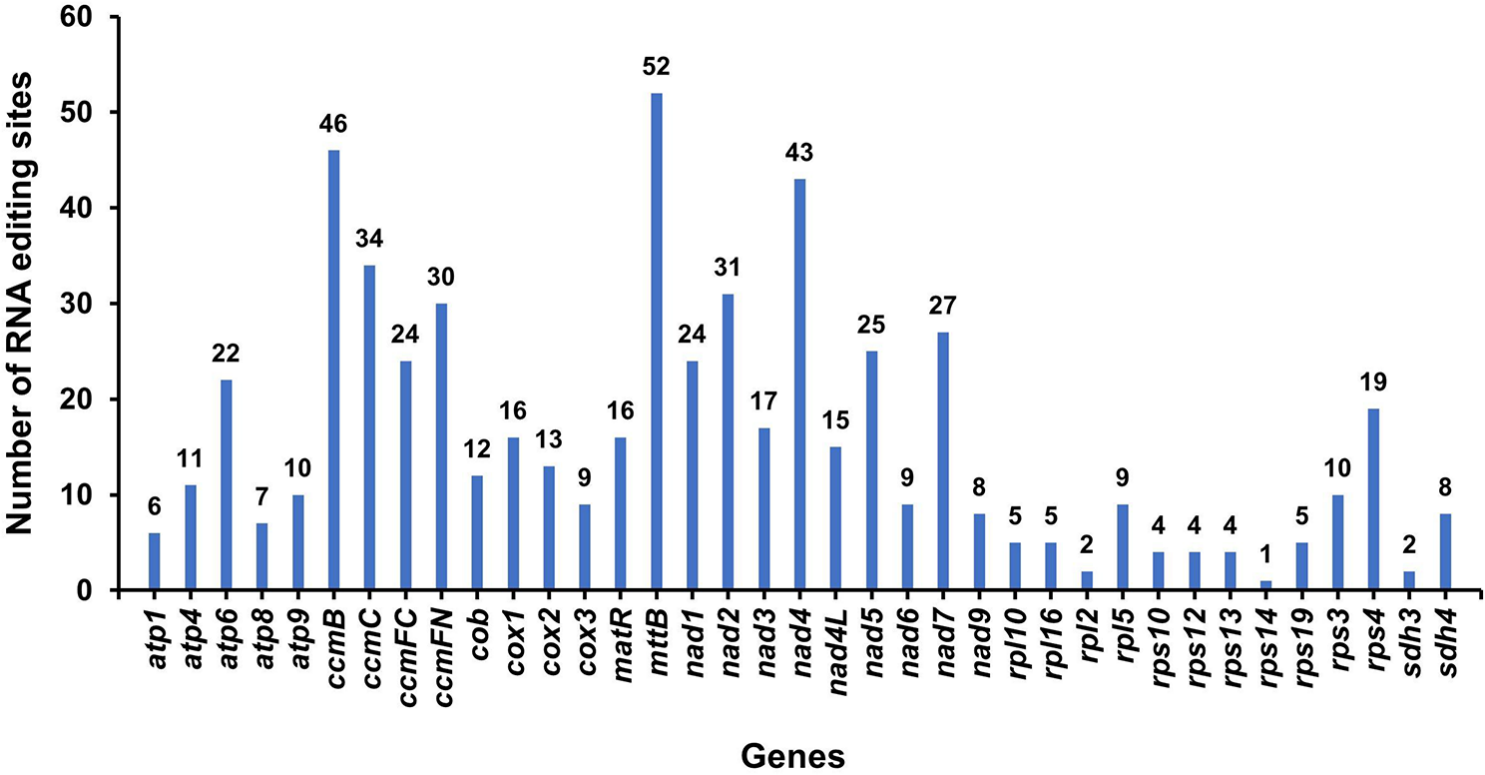
Number of RNA editing sites detected on each PCG in pepino mitochondria

RNA editing events were identified for 38 PCGs from pepino mitochondria based on RNA-seq data. In total, 585 potential RNA editing sites were identified on 38 mitochondrial PCGs (Table S11), and all involved C-to-U base editing. As shown in Fig. 8, *ccmB*, *ccmC*, *ccmFN*, *mttB*, *nad2*, and *nad4* were edited over 30 times, and *mttB* was edited 52 times, the most among all the genes. This was followed by *ccmB*, which exhibited 46 RNA editing events. However, *rps14* had undergone only one RNA editing event.

## Discussion

Mitochondria are the powerhouses of plants and produce the energy needed for plant growth and development [47]. It is now clear that the mt genome is a dynamically evolving entity that exhibits a complex diversity of genome size, structure, and gene content within a lineage or individually in plants [48–50]. This complex structure of the plant mt genome poses a great challenge for precision assembly [51–53]. In recent years, with the rapid development of sequencing technologies, numerous plant mt genomes have successfully been resolved. However, limited by the read length of next-generation sequencing data and the high error rate of third-generation sequencing data, the *de novo* assembly of complex plant mt genomes is challenging. PacBio HiFi sequencing integrates the advantages of high precision and long read lengths and is becoming the “gold” standard for the *de novo* assembly of plant mt genomes [54]. Herein, we completed the mt assembly for pepino using PacBio HiFi data and characterized the mt genome in detail for the first time. The complete mt genome of pepino consisted of one circular contig, which was 433,466 bp in length.

Previous studies have demonstrated that due to the presence of repeat sequences, plant mt genomes usually had multiple alternative or minor conformations [55–59]. In this study, we found that one pair of repeat sequences may have enabled the pepino mt genome to form two separate cyclic molecules (Fig. 1D). These phenomena may be the result of specific DNA repair mechanisms in plant mt genomes [60]. We verified the existence of these ring structures (Fig. 1B), but whether both ring molecules can coexist requires deeper study.

The repeat sequences included tandem, short, and large repeats, which are widespread in the mt genomes of higher plants [61, 62]. It is documented that repeats play a pivotal role in mitochondrial intermolecular recombination [63], especially long repeat sequences (> 1 kb), which may cause high-frequency recombination, leading to genomic isomerization into several major forms [64, 65]. In this study, the SSRs, tandem repeats, and dispersed repeats were investigated intensively (Fig. 4). Among the large number of repeat sequences identified, we found eight sequences exceeding 1 kb in length, ranging from 2,353 to 8,353 bp. The longer repeat sequences may have played a crucial role in shaping the pepino mt genome during evolution.

Transfer events from cp. to mt genomes occur frequently in angiosperms [66, 67]. Studies have revealed plastid-derived backgrounds containing 0.1–11.5% of mitogenomes [68]. In our study, we detected 33 MTPTs, 20,759 bp in length, which accounted for 4.79% of the *S. muricatum* mt genome. These fragments included 19 complete genes, namely eight PCGs and 11 tRNA genes (Table S8). Previous studies have revealed that the transfer of tRNA genes from the cp. to mt genome was common in angiosperms [59, 69, 70]. The 11 tRNA genes from the cp. genome of *S. muricatum* were complete genes (Table S8), suggesting they may play a role in normal functions [71].

Compared to plastid and nuclear genes, the PCGs of mt genomes are more suitable for exploring ancient diversity patterns than elucidating routine phylogenetic investigations in higher plants because of their slow mutation rate [72], frequent genomic rearrangement [73], and integration of foreign DNA from the nuclear and plastid genomes [74]. Previous studies have shown that pepino was closely related to tomato and potato phylogenetically, based on chloroplast DNA sequences data [41], time-calibrated phylogeny [42], transcriptomes, and genomes [43, 46]. In this study, the evolutionary relationships were well clustered between families and between genera (Fig. 6). However, the pepino was more closely related to tomato and more distantly related to potato (*Solanum*) (Fig. 6). This trend has also been detected in the mitogenomes of *Primula* [75] and *Avena longiglumis* [76]. Therefore, more mitochondrial assemblies are needed to analyze the evolutionary and phylogenetic implications of pepino mitochondria in detail.

RNA editing is widespread in the mt genomes of higher plants. The most prevalent RNA editing event is the post-transcriptional regulation of single base transitions, which plays vital roles in physiological processes and molecular functions [77, 78]. Previous studies have detected 491 and 441 RNA-editing sites the mt genomes of *Oryza sativa* and *Arabidopsis thaliana* [79, 80], respectively. Based on RNA-seq data, RNA-editing events in the pepino mt genome were identified; a total of 585 RNA editing sites were detected in 38 PCGs, all of which involved C-to-U editing (Fig. 8, Table S11). It has been reported that the start codons of many genes may be generated by RNA editing events. For example, the start codon of the *cox1* gene is generated by RNA editing of ACG to AUG in the mt genomes of potato and plum [77, 81]. Herein we also found this phenomenon in the mt genome of pepino. The mechanisms behind this need to be studied further.

## Conclusions

We assembled the mt genome of pepino for the first time using PacBio HiFi data. The mt genome of pepino, 433,466 bp in size with a GC content of 44.79%, included 38 PCGs, 19 tRNAs, and three rRNAs. Long reads and PCR verification revealed one pair of direct repeats (5,596 bp) in the pepino mt genome that promoted the rearrangement of the mitogenome to form a bicyclic structure. In addition, codon usage, sequence repeats, phylogenetic data, and synteny were analyzed. MTPT events were found in the cp. and mt genomes, suggesting that multiple transfer events may have occurred during the evolution of pepino. Subsequently, we used transcriptome data to detect the RNA editing sites of mt PCGs in detail and found them to be abundant, and all involved C to U editing. In conclusion, elucidating the mt genome of pepino will provide crucial information for evolutionary studies and lay a foundation for further molecular breeding of mitochondria-associated characters in pepino.

## Materials and methods

### Plant materials, DNA extraction, and sequencing

The tender leaves of pepino plants were collected from Shilin district, Kunming, Yunnan Province, China (Longitude: 103.64519, Latitude: 24.84990; altitude 2160 m). The plant sample was identified by Professor Hongzhi Wu in the Yunnan Agricultural University. The specimens of *S. muricatum* has been deposited at Herbarium of Kunming Institute of Botany, Chinese Academy of Sciences (voucher number: 1,589,547). The pepino leaves were kept at -80℃ until use. The total genomic DNA and RNA were extracted from ∼ 100 mg of frozen leaves utilizing a plant genomic DNA kit (TianGen Biotech, Beijing, China) and an RNAprep Pure Plant Kit (TianGen Biotech, Beijing, China), respectively. A Nanodrop spectrophotometer 2000 (Thermo Fisher Scientific, Waltham, MA, USA) was used to measure the DNA/RNA concentration. The purity of the DNA/RNA was evaluated using 1.0% agarose gel electrophoresis. The high quality DNA and RNA were placed on dry ice and sent to Wuhan GrandOmics Technology Co., Ltd. (http://www.grandomics.com) for PacBio (PacBio Sequel II platform; Pacific Biosciences, CA, USA) and Illumina (Illumina NovaSeq platform; Illumina, San Diego, CA, USA) sequencing.

### Genome assembly and annotation

First, *de novo* assembly of the pepino mitogenome was conducted using the PacBio HiFi data and PMAT software ( v1.0) with “autoMito” mode [82]. Secondly, the BLASTn (v2.2.30+) [83] program with the parameters “-evalue 1e-5 -outfmt 6 -max_hsps 10 -word_size 7 -task blastn-short” was used to identify the draft mt genome of pepino based on the assembled contigs. To obtain the draft mt genome, a conserved PCG database for the assembled contigs was constructed using makeblastdb, and then the 24 conserved plant mt PCGs conserved mt genes from *Arabidopsis thaliana* (NC_037304.1), *Brassica napus* (NC_008285.1), *Glycine max* (CM033153.1), *Populus alba* (NC_041085.1), *Nicotiana tabacum* (NC_006581.1), *Malus domestica* (OX352780.1), *Oryza sativa* (CP018169.1), *Sorghum bicolor* (NC_008360.1), *Triticum aestivum* (NC_036024.1), and *Zea mays* (CM059588.1) were used as a query sequence to identify contigs that contained conserved mt genes. Thereafter, we used Bandage software (v0.8.1) [84] to visualize the GFA files and manually remove “noisy” (chloroplast or nuclear contigs) and non-target contigs. Simultaneously, to resolve the repetitive regions in the obtained graphical genome, the largest single-copy fragment were selected as a starting point and exhaustively searches all possible paths using PMAT (v1.0) software [82] to generate the most likely mt genome structure. Finally, we obtained one circular contig for pepino.

The mitogenomes of *Arabidopsis thaliana* (NC_037304), *Liriodendron tulipifera* (NC_021152.1), *Solanum lycopersicum* (NC_035963.1), and *Solanum tuberosum* (MW594276_7.1) were used as the reference genomes, and Geseq (https://chlorobox.mpimp-golm.mpg.de/geseq.html) [85] was used to annotate the PCGs of the *S. muricatum* mt genome. In addition, tRNAs and rRNAs were detected using tRNAscan-SE software [86] (v2.0) (http://lowelab.ucsc.edu/tRNAscan-SE/) and BLASTn software [83], respectively. The annotation errors of each mt genome were corrected manually using Apollo software [87]. A circular diagram of the genome was drawn using OrganellarGenomeDRAW [88] (https://chlorobox.mpimp-golm.mpg.de/OGDraw.html).

### PCR amplification to confirm mitochondria genome structure

Based on the resolved conformation of the *S. muricatum* mt genome, we used Bandage software (v0.8.1) [84] to merge the pairwise connections into a single connected sequence. Then, Primer 5 software was used to design primers with a range of 1 kbp on either side of each node for each linkage variant. DNA was isolated from young leaf tissue using a DNA extraction kit (TianGen Biotech, Beijing, China) and used to conduct PCR verification. PCR amplification products that crossed linkage sites were then used to verify each linkage relationship (Table S2). The PCR amplification was performed with 1 µL of template, 0.5 µL of upstream and downstream primers, respectively, 10 µL of 2 × Taq Master Mix and 8 µL of ddH_2_O, with the following program: predenaturation at 94 °C for 2 min; denaturation at 94 °C for 30 s, annealing at 56 °C for 30 s, extension at 72 °C for 2 min, 35 cycles; and a final extension at 72 °C for 10 min. The PCR products were evaluated for length using a 1.0% agarose gel run at 120 V for 30 min and compared to a 5 kbp ladder.

### Analysis of RSCU and repeated sequences

The PCGs of the genome were extracted using Phylosuite [89]. Mega 7.0 [90] was used to conduct codon preference analysis for PCGs in the mitogenome and calculate RSCU values.

The repeat sequences, including microsatellite sequence repeats, tandem repeats, and dispersed repeats, were identified using MISA (v2.1) (https://webblast.ipk-gatersleben.de/misa/) [91], TRF (v4.09) (https://tandem.bu.edu/trf/trf.unix.help.html) [92], and REPuter web server (https://bibiserv.cebitec.uni-bielefeld.de/reputer/) [93]. The results were visualized using Excel (2021) software and the Circos package (v0.69-9) [94].

### Identification of mitochondrial plastid DNA (MTPT)

The chloroplast genome of *S. muricatum* was assembled and annotated using GetOrganelle (v1.7.6.1) [95] and CPGAVAS2 (v2.0) [96], respectively. The cp. genome annotation results were then corrected using CPGView (v1.0) software [97]. Thereafter, the reciprocal comparison strategy was used to identify the homologous fragments between the cp. genome and mt genome using BLASTN (v2.2.30+) [83] with the parameters “-evalue 1e-10-word_size 7-outfmt 6”. Finally, the results were visualized using the Circos package [94].

### Phylogenetic evolution and collinearity analysis

The 38 mt genomes of closely related species were used to construct a phylogenetic tree, with *Salvia miltiorrhiza* (NC_023209.1) and *Ajuga reptans* (NC_023103.1) from Lamiaceae as outgroups (Table S8). A total of 15 orthologous mt genes among the analyzed species were identified, extracted, and concatenated by PhyloSuite (v.1.2.1) [89]. The multiple sequences were aligned using MAFFT (v7.505) with the auto model [98]. Next, these aligned sequences were used to construct the phylogenetic trees. The maximum likelihood (ML) tree was constructed based on a GTR + F + I + G4 model using IQ-Tree (v2.1.4-beta) [99] with 1000 bootstraps. Finally, the ML tree was visualized using iTOL (https://itol.embl.de/) [100].

Data from seven mt genomes, including *Solanum bukasovii* (MW122985.1), *Solanum lycopersicum* (NC_035963.1), *Solanum pennellii* (NC_035964.1), *Solanum aethiopicum* (NC_050335.1), *Solanum melongena* (NC_050334.1), *Solanum sisymbriifolium* (MT122964_5.1), and *Solanum wrightii* (MT122958_9.1) from the Rosaceae family, were selected for the synteny analysis with *S. muricatum*. BLASTN [83] was conducted to compare eight mt genomes pairwise and obtain homologous sequences following the parameters: “-evalue 1e-5, -word_size 9, -gapopen 5, -gapextend 2, -reward 2, and -penalty 3”. Only conserved colinear blocks longer than 0.5 Kb were retained for subsequent analysis. The Multiple Synteny Plot of *S. muricatum* with the seven species was constructed based on sequence similarity using MCscanX [101].

### Detection of RNA editing sites

The RNA-editing sites were detected using the RNA-seq data sequenced in this study. The RNA-seq data were mapped to the PCGs with BWA [102] software to obtain transcripts from the mt genome. Differences between the DNA and RNA sequences were further compared with BEDTools software (v2.30.0) to identify RNA editing events supported by most reads, with the following cut-offs: coverage ≥ 5, frequency ≥ 0.1 and *P* ≤ 0.05 [103]. The results were visualized using Excel (2019) software.

### Electronic supplementary material

Below is the link to the electronic supplementary material.

Supplementary Material 1

## Data Availability

The raw sequencing data from the Illumina and PacBio platforms generated during the current study are available in GenBank. The associated BioProject and BioSample numbers are PRJNA1010652 and SAMN37193756, respectively. The Illumina and PacBio sequencing data of *S. muricatum* have been deposited in the GenBank repository under SRR25885158, SRR25885160, and SRR25885159, respectively. The chloroplast and mitogenome sequences have been submitted to GenBank with the following accession numbers: OR501824 and OR501825. The DNA sequences of *S. muricatum* mt genome and cp. genome were provided as fasta files, along with the annotation information, which is provided as Genbank files and is also available through Figshare at 10.6084/m9.figshare.24003855 and 10.6084/m9.figshare.24003885.
