# Supplementary material for: De novo assembly of the complete mitochondrial genome of pepino (Solanum muricatum) using PacBio HiFi sequencing: insights into structure, phylogenetic implications, and RNA editing
Source: BMC Plant Biol. 2024 May 4;24:361. doi: 10.1186/s12870-024-04978-w (PMC11069145; doi:10.1186/s12870-024-04978-w)
Supplement: Supplementary file 1 — Supplementary Material 1 [file 12870_2024_4978_MOESM1_ESM.docx]

Supplementary Information

The online version contains supplementary material available at https:// doi.org/ 10. 1186/ s12870- xxx- xxxxx-x.

Additional file 1: Table S1. Path selection for individual nodes (repetitive region) based on PacBio HiFi data. Table S2. Sequencing depth of coverage based on PacBio HiFi long-reads. Table S3. The primer designs to confirm the 4 connections of the mitochondrial genome conformation in *S. muricatum*. Table S4. Relative synonymous codon use of codons by individual amino acids in the *S. muricatum* mitochondrial genome. Table S5. SSRs in the mitochondrial genome of *S. muricatum*. Table S6. Tandem repeat sequences in the mitochondrial genome of *S. muricatum*. Table S7. Dispersed repeat sequences in the mitochondrial genome of *S. muricatum*. Table S8. The homologous DNA fragment in the S. muricatum mitochondrial genome. Table S9. The closed species with *S. muricatum*. Table S10. Analysis of collinearity between *S. muricatum* and related species. Table S11. RNA-editing in *S. muricatum* mitochondrial genome. Figure S1. Sequencing depth of coverage based on PacBio HiFi long-reads. Figure S2. The raw Gel diagram of agarose gel electrophoresis.


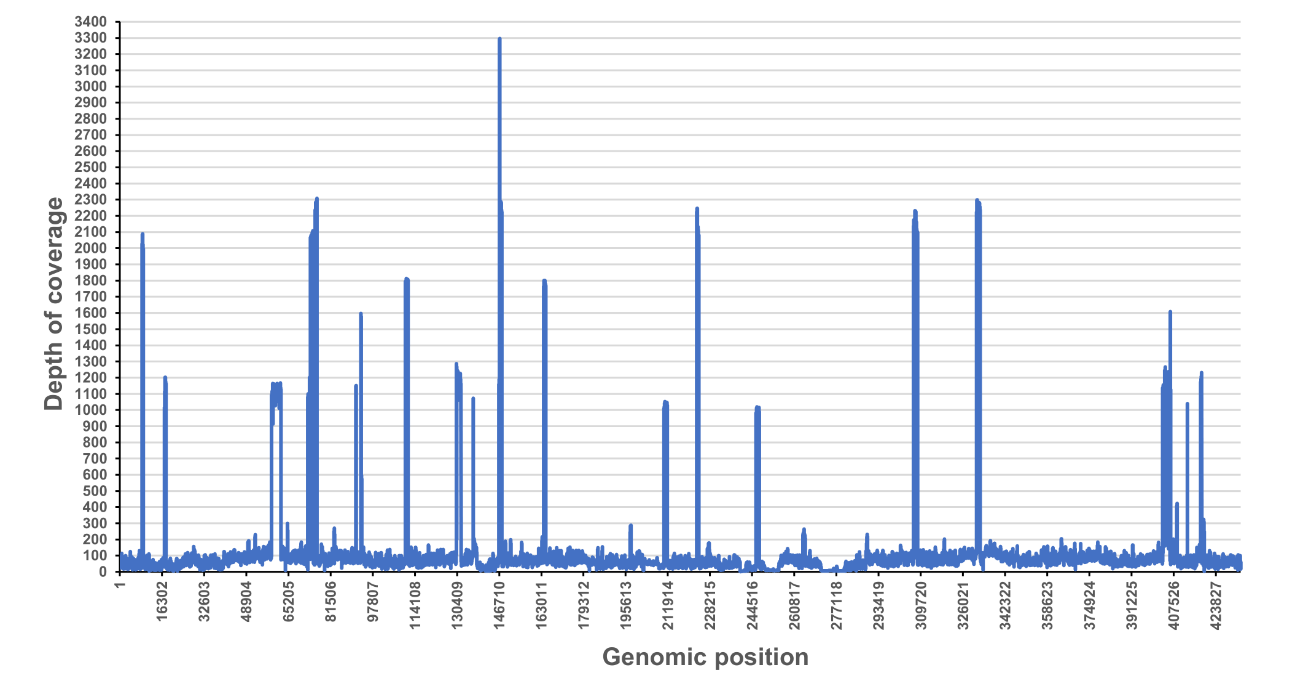


**Fig. S1** Sequencing depth of coverage based on PacBio HiFi long-reads. The abscissa indicates the location of the contig, and the ordinate indicates the sequencing depth. The average sequencing depth of the mitochondrial genome was about 147 ×.


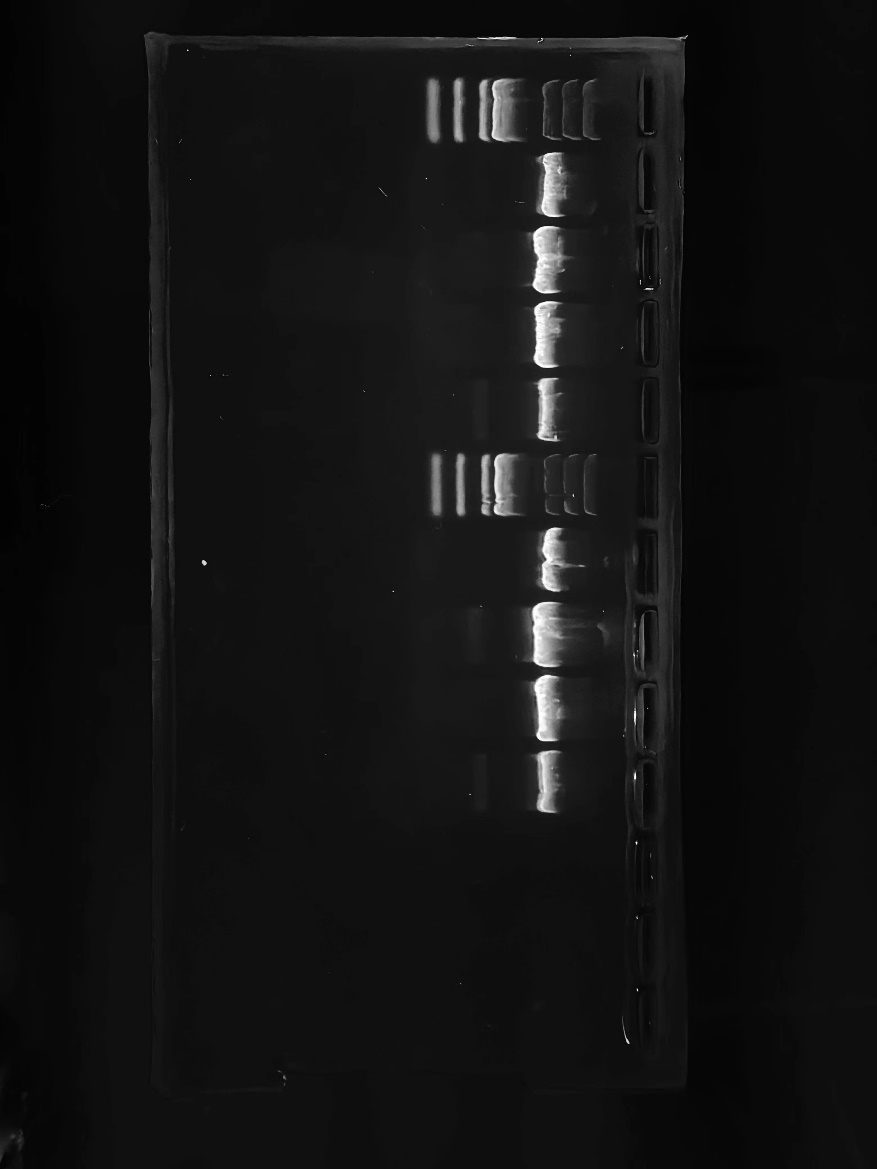


**left**

**right**

**5000bp**

**3000bp**

**2000bp**

**1000bp**

**750bp**

**500bp**

**250bp**

**100bp**

**Marker a b c d**

**Marker a b c d**

**Fig. S2** The raw Gel diagram of agarose gel electrophoresis. The numbers above each lane of the gel refer to linkages spanned by the primers with respect to the contig (a, b, c, and d represent the connections of ctg1-ctg3, ctg3-ctg1, ctg3-ctg2, and ctg2-ctg3, respectively).

Note: We've cropped the image on the left to place it in the manuscript.
